# Plasma thymidine kinase 1 activity and outcome of ER+ HER2− metastatic breast cancer patients treated with palbociclib and endocrine therapy

**DOI:** 10.1186/s13058-020-01334-2

**Published:** 2020-09-14

**Authors:** Luc Cabel, Dan Rosenblum, Florence Lerebours, Etienne Brain, Delphine Loirat, Mattias Bergqvist, Paul Cottu, Anne Donnadieu, Anne Bethune, Nicolas Kiavue, Manuel Rodrigues, Jean-Yves Pierga, Marie-Laure Tanguy, François-Clément Bidard

**Affiliations:** 1 Department of Medical Oncology, Institut Curie, Saint-Cloud, 92210 Paris, France; 2 Circulating Tumor Biomarkers Laboratory, SIRIC2 Institut Curie, Paris, France; 3 UVSQ, Université Paris-Saclay, Saint-Cloud, Paris, France; 4 Biovica, Uppsala, Sweden; 5 Université de Paris, Paris, France

**Keywords:** Metastatic breast cancer, CDK4/6 inhibitor, Palbociclib, Thymidine kinase 1, Biomarker

## Abstract

**Purpose:**

Previous cohort studies have reported plasma TK1 activity (pTKa) as a potential prognostic biomarker in estrogen receptor-positive (ER+) HER2-negative (HER2−) metastatic breast cancer (MBC). In this prospective study, we report here the prognostic impact of pTKa in ER+/HER2− MBC patients treated with endocrine therapy and CDK4/6 inhibitor.

**Experimental design:**

Patients were included into the prospective, ethics committee-approved ALCINA study (NCT02866149). Eligibility criteria were patients with ER+/HER2− MBC treated at Institut Curie with endocrine therapy and palbociclib. Plasma samples were obtained at baseline and after 4 weeks of treatment. pTKa was quantified by the DiviTum® assay (Biovica, Sweden).

**Results:**

From May 2016 to August 2018, 103 patients treated with endocrine therapy and palbociclib were included. Patients had received a median of two prior systemic therapies for MBC (range 0–14). Median follow-up was 13.8 months (range 6–31), with median PFS and OS of 9.6 months (95%CI [7.0–11.3]) and 28 months (95%CI [23–not reached]), respectively. Median baseline pTKa was 292 Du/L (range 20–27,312 Du/L, IQR [89–853]). After adjusting for other prognostic factors, baseline pTKa remained an independent prognostic factor for both PFS (HR = 1.3 95%CI [1.1–1.4], *p* = 0.0005) and OS (HR = 1.3 95%CI [1.2–1.6], *p* < 0.0001), and 4-week pTKa was associated with OS (HR = 1.6 95%CI [1.3–2], *p* < 0.0001). That survival prediction was significantly improved by the addition of baseline pTKa to clinicopathological characteristics. Adding pTKa changes at 4 weeks to baseline pTKa did not further increase survival prediction.

**Conclusion:**

This study demonstrates the clinical validity of pTKa as a new circulating prognostic marker in ER+/HER2− MBC patients treated with endocrine therapy and palbociclib.

## Introduction

Endocrine therapy is the cornerstone of treatment of estrogen receptor-positive (ER+) HER2-negative (HER2−) breast cancer [1]. Endocrine therapies for metastatic breast cancer (MBC) have remained largely unchanged for the past two decades. They include tamoxifen, aromatase inhibitors (AI), and fulvestrant. More recently, CDK4/6 inhibitors (palbociclib, ribociclib, abemaciclib) have been shown to be effective in ER+ HER2− MBC as first-line therapy in combination with letrozole [2–4] or fulvestrant (a selective ER degrader) after progression on AI [5, 6]. However, no biomarker is currently validated to guide the use of these new drugs in clinical practice.

Thymidine kinase 1 (TK1) plays a crucial role in DNA synthesis and cell proliferation [7, 8]. In preliminary studies using the same assay, serum or plasma TK1 activity (sTKa, pTKa) was reported to be a prognostic circulating biomarker in early and advanced breast cancer [9–12]. The prognostic impact of baseline serum TK1 levels and TK1 variation in response to therapy was also reported in 159 samples from a phase III trial comparing AI and fulvestrant in ER+ HER2− MBC [13].

We report here the prognostic impact of pTKa, assessed in a prospective study on ER+ HER2− MBC patients treated with endocrine therapy and CDK4/6 inhibitor.

## Patients and methods

### Study

After providing their written informed consent, patients were prospectively included in the ethics committee-approved ALCINA study (NCT02866149), investigating the feasibility of analysis of circulating tumor markers in blood in various early- or advanced-stage malignancies. Eligibility criteria were patients aged > 18 years with ER+ HER2− MBC, treated at Institut Curie with endocrine therapy (AI or fulvestrant) and palbociclib. Blood samples were obtained at baseline (prior to treatment start) and after 4 weeks of treatment.

### Blood collection and pTKa assessment

At each time point, 21 mL of blood was drawn in STRECK tubes and rapidly processed (< 72 h) to obtain 6–8 mL of plasma after centrifuging blood at 820 g for 10 min. Plasma was transferred to 2 mL tubes and centrifuged at 16,000*g* for 10 min to remove debris and stored at − 80 °C until needed.

pTKa was determined by the DiviTum® assay, a refined ELISA-based method, at Biovica laboratories in Uppsala, Sweden. Analysis was performed with no access to, or any knowledge of, patient or tumor characteristics. Each sample was diluted to 1/10 in dilution buffer and then incubated with a reaction mixture on the assay microtiter plate. Bromodeoxyuridine (BrdU), a thymidine analog, was phosphorylated to BrdU-monophosphate by the TK present in the sample, then further phosphorylated and incorporated in a DNA strand bound to the bottom of the wells. BrdU incorporation was detected by an ELISA technique using anti-BrdU monoclonal antibody conjugated to alkaline phosphatase and a chromogenic substrate, producing a yellow reaction product. Absorbance was measured at 405 nm with the reference wavelength of 630 nm after 30 and 60 min of incubation. The measured optical density is proportional to the TK enzymatic activity (TKa) of each sample, expressed as DiviTum® units per liter (Du/L), calculated from a standard curve based on calibrators of known activity [14].

### Statistical analyses

The study had no prespecified power. Associations between baseline pTKa and patients’ characteristics were studied with the Mann-Whitney test. OS and PFS were estimated with the Kaplan-Meier method. The association between pTKa (baseline or after 4 weeks) and survival end points was studied using restricted cubic splines with 3 knots. We used a square-root transformation due to the highly right-skewed pTKa distribution. The log-linearity of the relationship between the hazard ratio and pTKa was tested by using the Wald test for nonlinear components of restricted cubic splines. No evidence of nonlinearity was found.

The choice between modeling pTKa as a continuous variable (splines) or as a binary variable (median cutoff) was guided by the Akaike’s Information Criterion of univariate Cox models. The survival curves according to the median cutoff are presented for illustrative purposes.

OS and PFS according to the other prognostic factors (including the variation of pTKa, dichotomized as increase of more than 10% from baseline vs other) were compared in univariate analysis by means of a two-sided log-rank test. Variables with a *p* value ≤ 0.2 in univariate analysis were included in a multivariable Cox model with backward procedure for selecting covariates to be kept in the final model.

The correlation between baseline and pTKa at week 4 was studied with the Spearman rank correlation. All reported *p* values are two-sided.

## Results

### Patients

From May 2016 to August 2018, 103 patients treated with endocrine therapy (fulvestrant (*N* = 93, 90%) or AI (*N* = 10, 10%)) and palbociclib were included. Clinicopathological characteristics are described in Table 1. Median age was 64 years (range 26–89). Patients had received a median of two previous lines of therapy in metastatic setting (range 0–14); most patients presented a good performance status (92% with PS 0–1) and 66% had visceral metastasis. Seventeen patients (17%) presented primary resistance to endocrine therapy (defined according to ABC4 guidelines [1].

**Table 1:** Patients characteristics and association with baseline pTKa

Characteristics	***N*** (%)	Median TKa Du/L [IQR]	***p*** value
**Age**			
< 65 years	53 (51)	455 [117–1325]	0.2
≥ 65 years	50 (49)	233 [70–705]	
**Performance status**			
0–1	95 (92)	320 [86–865]	0.9
≥ 2	8 (8)	247 [150–789]	
**Grade**			
1	15 (15)	482 [99–771]	0.8
2	61 (61)	292 [117–917]	
3	24 (24)	286 [68–710]	
Missing value	3		
**Progesterone receptor**			
Negative	29 (28)	436 [117–1361]	0.1
Positive	74 (72)	237 [73–546]	
**Synchronous metastasis**			
No	71 (69)	195 [85–640]	0.1
Yes	32 (31)	476 [111–1292]	
**Visceral metastasis**			
No	35 (34)	249 [116–483]	0.6
Yes	68 (66)	327 [87–1033]	
**Primary endocrine resistance**			
No	86 (83)	237 [73–588]	**0.04**
Yes	17 (17)	758 [233–1641]	
**Previous chemotherapy**			
No	48 (47)	214 [61–781]	0.4
1 or 2 lines	34 (33)	330 [120–704]	
≥ 3 lines	21 (20)	466 [157–1281]	

All tumors were estrogen receptor-positive

*TKa* thymidine kinase activity, *IQR* interquartile range

With a median follow-up of 13.8 months (range 6–31), 73 relapses (71%) and 25 deaths (24%) were observed, with a median PFS of 9.6 months (95%CI [7–11.3]) and a median OS of 28 months (95%CI [23–not reached]).

### Association between baseline pTKa and clinicopathological characteristics

Median baseline pTKa was 292 Du/L (range 20–27,312 Du/L, IQR [89–853]). Associations between baseline pTKa and patient characteristics are shown in Table 1. Among patient characteristics, a significant association was observed between baseline pTKa and endocrine resistance status: patients with primary endocrine resistance displayed higher pTKa (median 758 Du/L versus 237 Du/L, *p* = 0.04). The proliferation marker Ki67 expression level, assessed on primary tumors, was available for 61 patients and was not correlated with baseline pTKa (Spearman *r* = 0.03, *p* = 0.8).

### pTKa and outcome

**Fig. 1:**
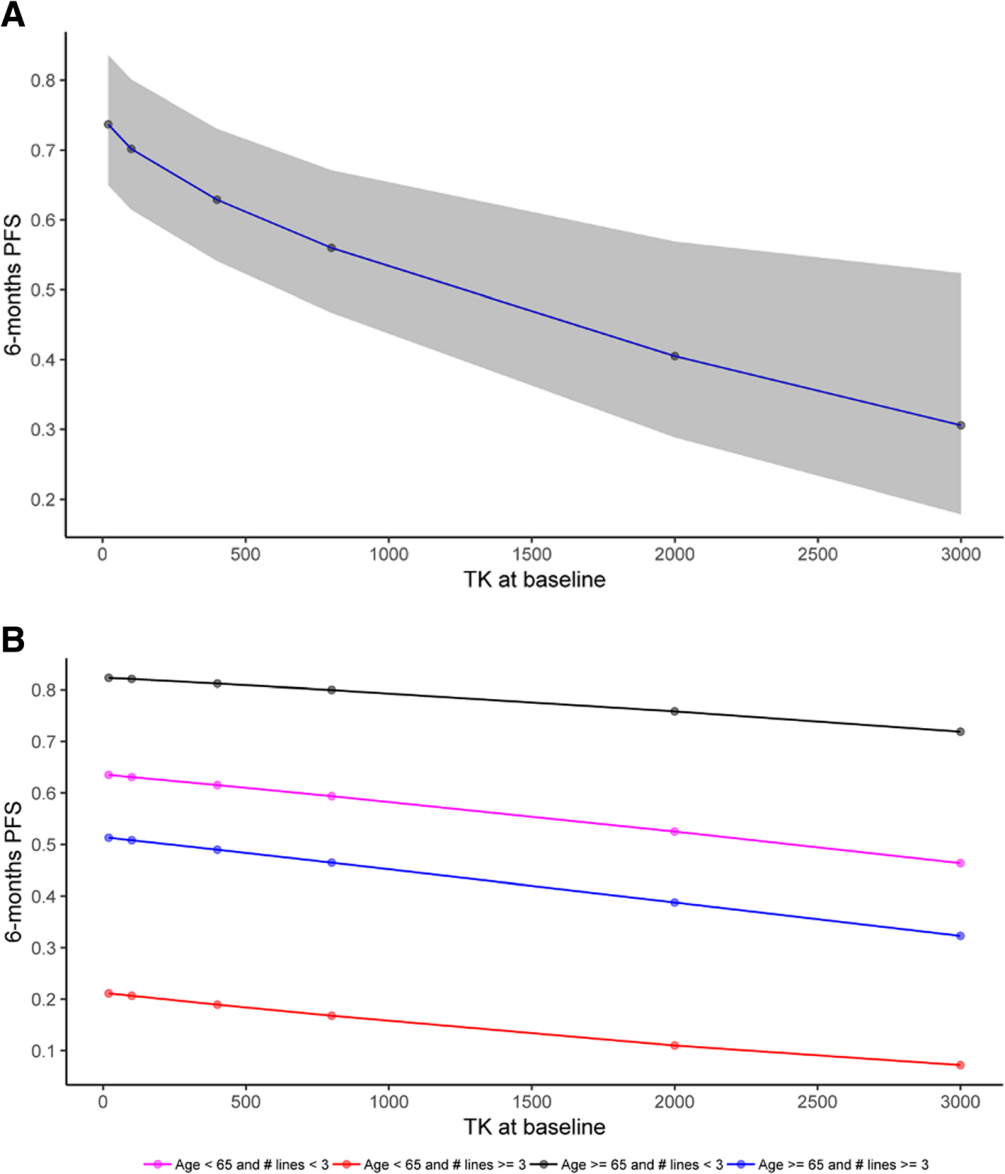
6-month PFS according to baseline TKa (with 95%CI) predicted by univariate model (**a**) and by multivariate model according to age or number of lines of chemotherapy (**b**)

**Fig. 2:**
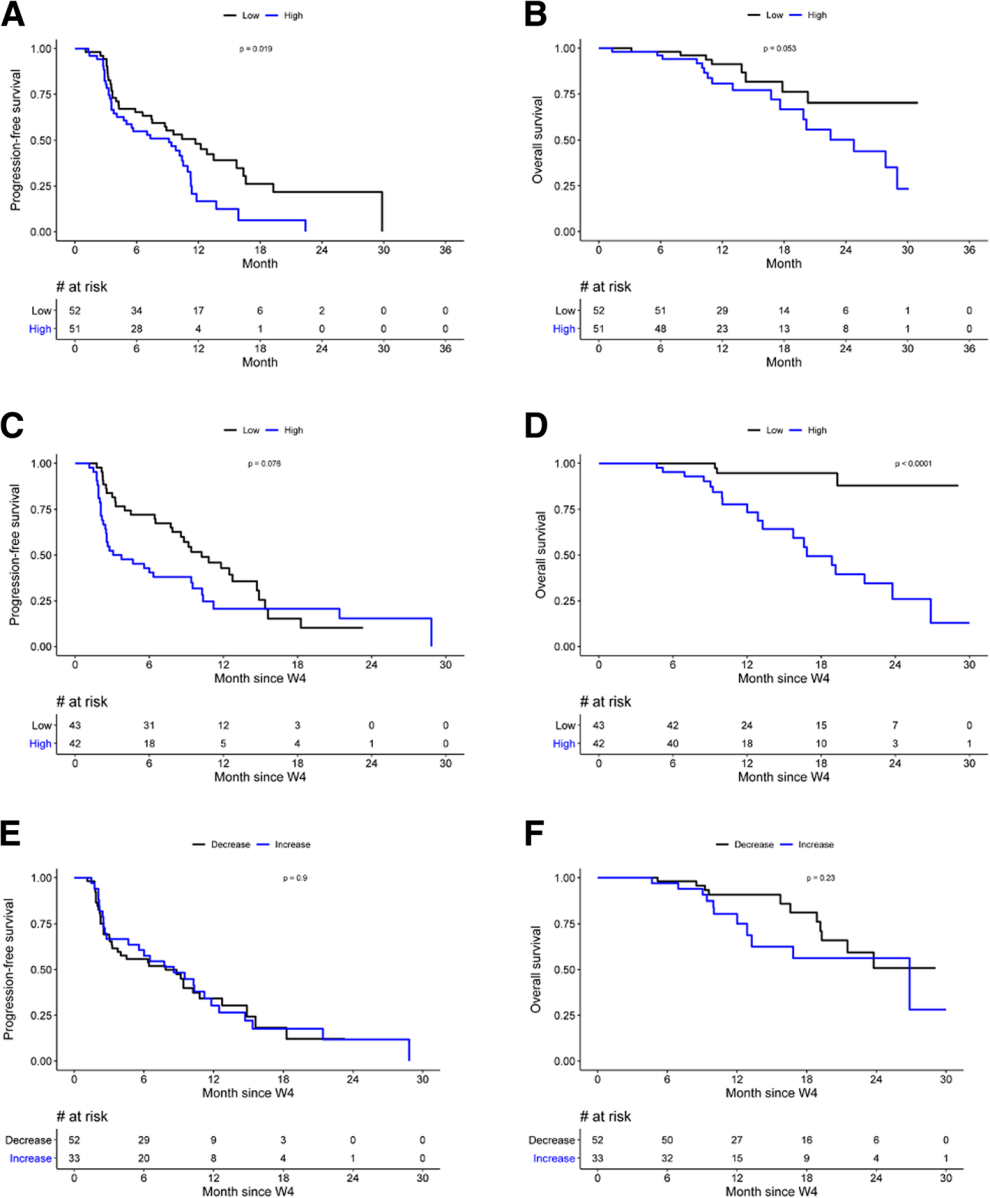
Progression-free survival and overall survival according to median pTKa levels: **a** PFS according to Baseline pTKa (cutoff 292 Du/L). **b** OS according to Baseline pTKa. **c** PFS according to 4 weeks pTKa (cutoff 134 Du/L). **d** OS according to 4 weeks pTKa. **e** PFS according to pTKa level changes between baseline and 4 weeks. **f** OS according to pTKa level changes between baseline and 4 weeks

Baseline pTKa (treated as a continuous variable) was significantly associated with both PFS and OS in univariate analysis (Table 2)**,** with HR = 1.3 95%CI [1.2–1.5]; *p* < 0.0001 and HR = 1.4 95%CI [1.2–1.6]; *p* = 0.0006, respectively. PFS (and OS) decreases with increasing pTKa (HR = 1.3, indicating a 30% increase of the hazard of progression or death when $\sqrt{\mathrm{pTKa}\ }$ increases of 10 units). To illustrate the link between PFS and TKa, Fig. 1a shows the predicted 6-month PFS according to baseline pTKa. Survival curves for dichotomized baseline pTKa according to median cutoff are shown in Fig. 2a and b (median PFS 11.7 months (95%CI [7.4;16]) vs 9.1 months (95%CI [4.4;14]), median OS not reached vs 24.8 months 95%CI [16–33]).

**Table 2:** Prognostic factors for progression-free survival (PFS) and overall survival (OS) in univariate analysis

	12 months PFS rate [95%CI]	***p*** value	12 months OS rate [95%CI]	***p*** value
**Age (years)**				
< 65	24.6 [14.1–43.1]	**0.01**	83.8 [73.4–95.7]	0.3
≥ 65	30.8 [17.9–53]		89 [80.3–98.75]	
**Performance status**				
0–1	36.7 [27.5–49]	0.5	88.8 [82.1–96]	**0.05**
2	0		56.2 [28.1–100]	
**Grade**				
< 3	38.1 [27.6–52.6]	0.2	85.4 [76.9–94.8]	0.7
≥ 3	23.2 [10.9–49.1]		86.8 [74–100]	
**PR status**				
Negative	35.7 [21.4–59.5]	0.5	80.3 [66.1–97.6]	0.1
Positive	33.8 [23.5–48.7]		88.9 [81.3–97.1]	
**Synchronous metastasis**				
No	37.7 [27–52.7]	**0.05**	95.3 [90.3–100]	0.07
Yes	27.8 [15–51.4]		68 [52.7–87.9]	
**Visceral metastasis**				
No	39.5 [25.3–61.6]	0.6	80.4 [67.3–96]	0.7
Yes	27.8 [15–51.4]		89.5 [81.8–97.9]	
**Primary endocrine resistance**				
No	40.4 [30.3–53.8]	**0.02**	90.2 [83.5–97.5]	**0.01**
Yes	11.8 [3.2–43.2]		69 [49.7–95.8]	
**Previous chemotherapy**				
No	49.7 [36.2–68.4]	**< 0.001**	100% *-* No death	**0.01**
1 or 2 lines	35.1 [21.1–58.2]		76.6 [62.8–93.6]	
≥ 3 lines	4.8 [0.7–32.2]		74.8 [57.8–96.7]	
pTKa at baseline*	HR = 1.3 [1.2–1.5]	**< 0.0001**	HR = 1.4 [1.2–1.6]	**0.0006**
pTKa at 4 weeks*	HR = 1.3 [1.1–1.5]	**0.01**	HR = 1.5 [1.2–1.8]	**0.0003**
**Variation of pTKa between baseline and 4 weeks**				
Increase	30.4 [17.6–52.3]	0.9	75 [60.1–93.7]	0.2
Decrease	34.2 [22.6–51.6]		90.7 [82.4–99.9]	

*HR for an increase of 10 units of $\sqrt{\mathrm{pTKa}}$

Other baseline prognostic factors in univariate analysis were primary endocrine resistance, number of lines of chemotherapy and performance status for OS and primary endocrine resistance, number of lines of chemotherapy, age, and metastasis-free interval for PFS (Table 2).

In multivariate analysis, baseline pTKa remained significantly associated with PFS and OS with HR = 1.3 95%CI [1.1–1.4], *p* = 0.0005, and HR = 1.3 95%CI [1.2–1.6] *p* < 0.0001, respectively (Table 3). In multivariate analysis, age <  65 years (HR = 2 95%CI [1.2–3.2]; *p* = 0.007) and ≥ 3 lines of chemotherapy (HR = 3.2 [1.8–5.7]; *p* < 0.0001) remained significantly associated with PFS. PS score (HR = 4.3 95%CI [1.3–13.5]; *p* = 0.01), ≥ 3 lines of chemotherapy (HR = 3.1 95%CI [1.3–7.6]; *p* = 0.01) and the presence of synchronous metastasis (HR = 3.1 95%CI [1.3–7.5]; *p* = 0.01) remained significantly associated with OS. The predicted 6-month PFS according to the prognostic factors identified in the multivariate model is presented in Fig. 1b.

**Table 3:** Prognostic factors for progression-free survival (PFS) and overall survival (OS) in multivariate analysis

	Baseline			
	PFS		OS	
Adverse prognostic factor	HR [95%CI]	***p*** value	HR [95%CI]	***p*** value
**Age** < 65 years	2 [1.2–3.2]	0.007	N.S	
**≥ 3 lines of chemotherapy**	3.2 [1.8–5.7]	< 0.0001	3.1 [1.3–7.6]	0.01
Baseline pTKa*	1.3 [1.1–1.4]	0.0005	1.3 [1.2–1.6]	< 0.0001
**Performance status = 2**	N.S	N.S	4.3 [1.3–13.5]	0.01
**Synchronous metastasis**	N.S	N.S	3.1 [1.3–7.5]	0.01

*HR for an increase of 10 units of $\sqrt{\mathrm{pTKa}}$

pTKa was available after 4 weeks of treatment for 85 patients (82.5%): median pTKa was 134 Du/L (range 20–3877 Du/L, IQR [43–595]) and was moderately correlated with baseline pTKa (*r* = 0.6, *p* < 0.0001). pTKa at 4 weeks was significantly associated with both PFS and OS in univariate analysis with HR = 1.3 95%CI [1.1–1.5], *p* = 0.01, and HR = 1.5 95%CI [1.2–1.8], *p* = 0.0003, respectively (Table 2).

PFS and OS according to pTKa at 4 weeks dichotomized between low (< median, 134 Du/L) and high (> median) are presented in Fig. 2c and d.

Although pTKa at 4 weeks was significantly associated with OS (HR = 1.6 95%CI [1.3–2]; *p* < 0.0001) in a multivariate model including the others clinical prognostic factors, when both pTKa values (baseline and 4 weeks) were included in the model, only baseline pTKa remained significant. pTKa at 4 weeks was not associated with PFS in multivariate analysis (regardless of the inclusion of the baseline value in the model).

**Fig. 3:**
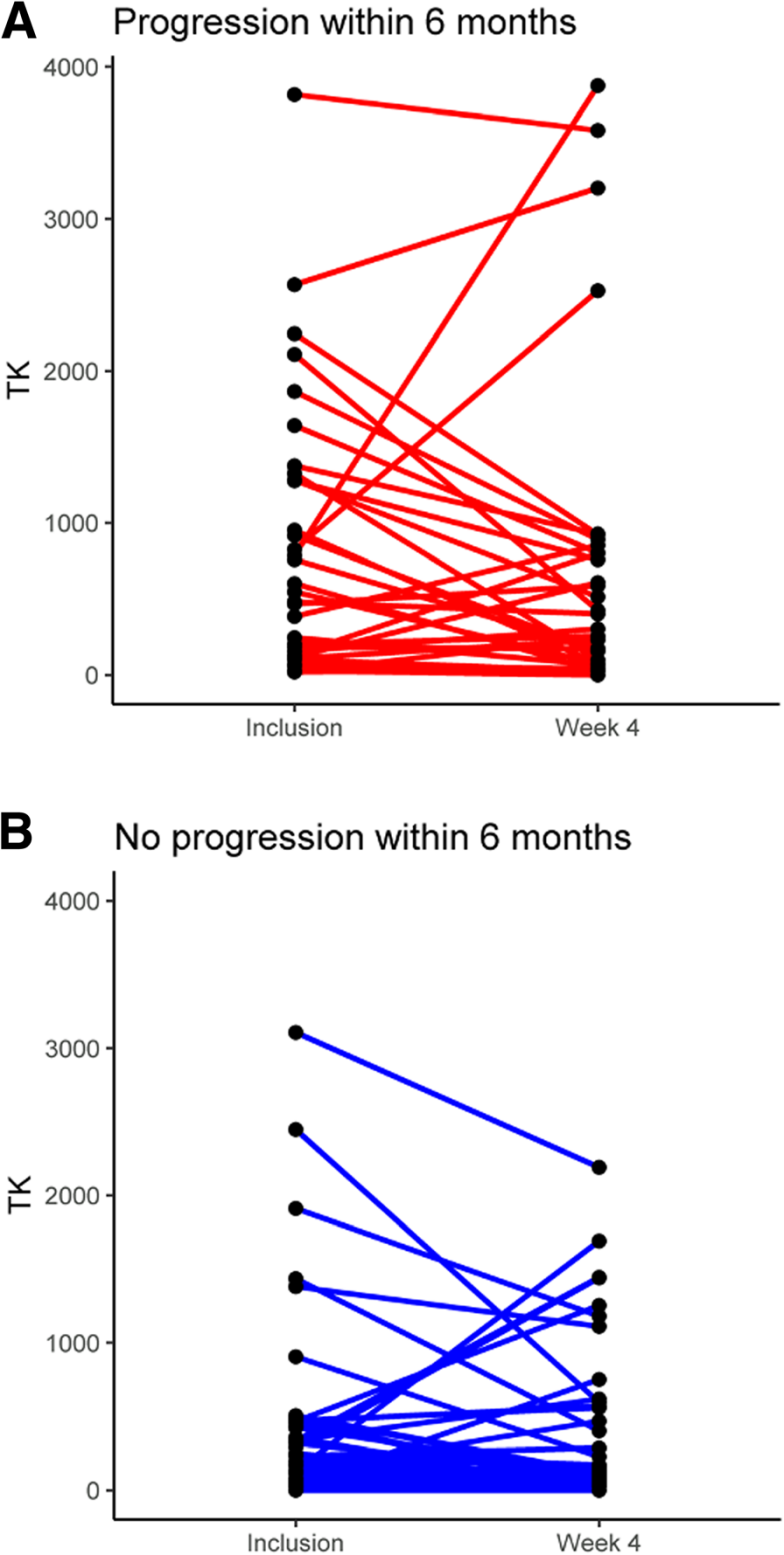
pTKa variation between baseline and 4 weeks for the progression group (**a**) or progression-free group (**b**)

The variation of pTKa between baseline and 4 weeks had no prognostic impact on either PFS or OS (Table 2, Fig. 2e, f), with a 12-month PFS of 34.2% when pTKa decreased versus 30.4% when pTKa increased (*p* = 0.9), and a 12-month OS of 90.7% 95%CI [82.4–99.9] versus 75% 95%CI [60.1–93.7] (*p* = 0.2), respectively. pTKa variation between baseline and 4 weeks according to 6-month progression-free survival is shown in Fig. 3. The median decrease of pTKa was 49 Du/L in the progression group (PFS < 6 months, *N* = 35) versus 23 Du/L in the progression-free group (PFS ≥ 6 months, *N* = 50) (*p* = 0.003).

## Discussion

To our knowledge, this is the largest study in ER+ HER2− MBC demonstrating the prognostic impact of pTKa at baseline and at 4 weeks in patients treated with endocrine therapy plus CDK4/6 inhibitors.

TK1 is an enzyme that is active during the cell cycle, especially during DNA synthesis. Its primary role is to catalyze thymidine into thymidine monophosphate. A low level of TKa is observed in resting cells, but TKa starts to increase during G1/S phase to reach its highest level during S phase and then disappears at mitosis. Several studies have shown that TKa is higher in cancer patients than in the healthy population [15], including in patients with breast cancer [14, 16].

Previous studies in BC have demonstrated a correlation between KI67 and serum TKa [17]. In ER+ HER2− BC treated with anastrozole and palbociclib in the neoadjuvant setting, Bagegni et al. showed that the sensitivity and specificity of the decrease in serum TKa to predict tumor Ki-67 reduction in response to palbociclib were 94.1% (95%CI [86.2–100%]) and 84% (95%CI [69.6–98.4%]), respectively. As Ki67 is known to be a biomarker of tumor response at 15 days from initiation of ET but requires a tumor biopsy, determination of pTKa could be a non-invasive way of measuring this response. In the present study, we did not observe any association between TKa and tumor Ki67 or SBR grade, which could be explained by the time-lag between determination of Ki67 or SBR grade, assessed on the primary tumor, and determination of pTKa in an advanced metastatic setting (as no tumor biopsy of metastasis was available at the start of ET+ CDK4/6 treatment).

Plasma or serum TKa has been shown to be a prognostic factor in other metastatic cancers [18–20]. Several studies have been conducted in various MBC settings using the same assay (DiviTum) [21]. In 198 MBC patients (mostly HER2-negative) treated with chemotherapy, Bjöhle et al. showed that pretreatment serum TKa predicted PFS and OS in multivariate analysis (OS, HR = 1.81, 95%CI [1.26–2.61], *p* = 0.001, with a cutoff of 235 Du/L) [22]. Larsson et al. also demonstrated a significant association between pTKa and PFS and OS in univariate and multivariate analyses at baseline and at 1, 3, and 6 months in women with MBC receiving first-line systemic therapy [23]. In ER+ HER2− MBC treated with ET only, sTKa was shown to be a prognostic factor at baseline and during treatment in the EFECT trial [13], in which the median time to progression for patients (*N* = 111) with low baseline serum TKa levels (<  97 Du/L) was 5.03 months (95% confidence interval [CI] 3.9–5.9) versus 2.57 months (95%CI 2.0–3.5) in patients with high baseline serum TKa levels (*p* < 0.0001). A smaller cohort of 31 patients, mostly previously untreated for MBC, also showed a prognostic value of pTKa for PFS with a cutoff of 122 Du/L [24]. We have confirmed the prognostic impact of pTKa on PFS and OS in MBC ER+ HER2− patients treated with ET + CDK4/6, both at baseline and at 4 weeks, even if baseline pTKa remained the most powerful prognostic factor at 4 weeks. Thus, the 4-week value seems to have less interest when the baseline has been obtained.

The EFECT trial [13] suggested that patients whose sTKa increased from baseline while on endocrine therapy had a significantly shorter time to progression (3.39 months, 95%CI 2.14–4.11) than those in whom sTKa did not increase (5.39 months, 95%CI 4.01–6.68) (*p* = 0.0045). More recently, the TREnd trial [25] showed a significant correlation between the 4-week variation of pTKa and PFS in ER+/HER2− MBC patients treated with palbociclib (alone or in combination with endocrine therapy following progression during a previous line of therapy). In the TREnd study, pTKa increase (*n* = 33 patients) was associated with shorter PFS (median PFS for the group with increasing pTKa = 3.0 months, 95%CI [2.7-NA] versus 9 months for stable or decreasing pTKa, 95%CI [5.8–12.0], *p* = 0.002). Bonechi et al. also showed that patients with decreasing pTKa activity after 1 month of treatment had a significantly better PFS comparatively to the increasing pTKa group (14.5 months versus 3.8 months, respectively, *p* = 0.0026) [24]. In contrast, no significant association was observed between baseline pTKa and PFS. In the present study, we did not find a predictive impact of the variation of pTKa between baseline and 4 weeks, regardless of the increase/decrease cutoff used (data not shown), unlike these other studies [13, 24, 25]. These contradictory results could be explained by several factors. First, the patient population, particularly with regard to the number of lines of chemotherapy received at the time of inclusion. In our study, 20% of patients received at least 3 lines of chemotherapy and 33% received 1 or 2 lines. In constrast the patients included in the TREnd trial had received a maximum of only one line (18%) and those in Bonechi’s study were mostly chemotherapy-naive (67.7%) or had a maximum of 2 lines. Thus, patients in our study appeared to be at a more advanced stage of disease, with tumors becoming resistant to multiple lines of treatment, leading to increased cell proliferation. This might explain a higher median pTKa at baseline compared to the other studies (292 Du/L vs 75–122 Du/L in other studies), although the rate of visceral metastases was globally similar. Because of these differences in median pTKa across studies and clinical settings, a pTKa threshold should be defined according to treatment history and using a large and homogeneous population, for a better assessment of the prognostic impact of baseline pTKa and pTKa variation. Importantly, chemotherapy having been reported to increase TKa levels [26, 27], the early interpretation of the variation could be challenging and this might explain that the variation of pTKa level was not a prognostic factor here. Ongoing large studies like SWOG S0226 (NCT00075764), resistance to first-line therapy with an AI and palbociclib at Johns Hopkins (NCT03439735), and the PYTHIA study (NCT02536742) will address this. Another difference between our study and previous studies was the number of patients included in these studies, which was lower in Bonechi’s study (*N* = 29) and TREnd (*N* = 41) than in our study (*N* = 85). Concerning the timepoint, in the EFECT trial, the second time point was collected at 3 months and not at 4 weeks, which could also explain a discrepancy with this study.

The limitations of this study include the lack of power and the heterogeneity of patients, especially in terms of the number of previous lines of ET+ CDK4/6 inhibitor therapy, making it difficult to compare pTKa cutoffs to predict outcome between the various studies. The results of prospective studies with a homogeneous cohort, receiving first-line or second-line CDK4/6 inhibitors are eagerly awaited (NCT03439735 and NCT02536742), as well as an interventional study to define patients in which ET+ CDK4/6 inhibitors should be intensified or could be de-escalated in the case of poor tolerability while maintaining a good prognosis.

## Conclusion

This study demonstrates the clinical validity of pTKa as a new circulating prognostic marker for PFS and OS in ER+ HER2− MBC patients treated with endocrine therapy and palbociclib. The clinical utility of this promising biomarker needs to be determined in an adequately designed prospective trial of combined targeted and endocrine therapies.

## Data Availability

All the data supporting the results of this manuscript are available from the corresponding author on request.
